# Evaluation and Comparison of the Precision of Dental Implant Impressions and the Marginal Fit of Crowns Using Conventional and Digital Methods: A Clinical Study

**DOI:** 10.7759/cureus.89415

**Published:** 2025-08-05

**Authors:** Bharat Joshi, Manne Prakash, Haragopal Surapaneni, Samyuktha Devarapalli, Mounika Checka, Durga P Konapala, Seema Gupta

**Affiliations:** 1 Department of Prosthodontics, Sibar Institute of Dental Sciences, Guntur, IND; 2 Department of Orthodontics, Kothiwal Dental College and Research Centre, Moradabad, IND

**Keywords:** conventional, digital, impression, intraoral scanner, marginal fit, precision

## Abstract

Background

Accurate impressions are essential for the long-term success of implant-supported restorations. Both conventional and digital techniques are routinely used in clinical implantology, each with its own advantages and limitations. This study aimed to evaluate and compare the precision of digital and conventional implant impressions and compare the marginal fit of crowns obtained from digital impressions and those obtained from conventional impressions.

Methodology

This prospective, comparative, clinical study was conducted at the Department of Prosthodontics between January and December 2024. A total of 36 subjects with single-unit implants were included in this study. Digital impressions (n = 18) were obtained using a calibrated intraoral scanner (3Shape Trios 4, Copenhagen, Denmark), and conventional impressions (n = 18) were made with polyvinyl siloxane using a closed-tray technique and scanned using a laboratory scanner (Medit T310 D, Medit Corp., Seoul, South Korea). Both generated Standard Tesselation Language files were analyzed for precision using Geomagic Control 2014 version 2.0 software (3D Systems, Rock Hill, SC, USA) via best-fit alignment. The crowns were designed in Exocad, fabricated via direct metal laser sintering, and assessed for marginal fit using the silicone replication technique under a stereomicroscope at 40× magnification. Data were analyzed using one-way analysis of variance for intragroup comparisons and independent t-tests for intergroup comparisons (p < 0.05).

Results

Precision analysis indicated no significant difference between the conventional and digital methods (p = 0.672), despite the greater variability in the digital group. Intragroup analysis revealed no significant differences in marginal discrepancy across the four sites (distobuccal, mesiobuccal, distolingual, and mesiolingual) between the conventional and digital groups (p > 0.05). Intergroup comparisons revealed no significant differences in marginal discrepancies between the groups at all sites (p > 0.05).

Conclusions

Conventional and digital impression techniques demonstrated comparable precision and marginal fit for single-unit implant restorations and achieved clinically acceptable outcomes. Further research is warranted to explore multi-unit restorations and long-term clinical outcomes.

## Introduction

The evolution of dental technology has significantly transformed restorative dentistry, particularly that of implant-supported prostheses. The precision of implant impressions and marginal fit of crowns are critical factors that influence the long-term success of implant restorations [1]. Conventional impression techniques involving elastomeric materials and physical casts have traditionally been the standard for capturing implant positions and fabricating restorations [2]. However, these methods are susceptible to errors stemming from material distortion, tray deformation, or operator variability, which may compromise the accuracy of the final prosthesis [3]. In recent years, digital impression techniques utilizing intraoral scanners have emerged as promising alternatives that offer potential advantages in terms of efficiency, patient comfort, and precision [3,4].

These systems capture three-dimensional (3D) images of the oral environment, which are then processed to design and fabricate restorations using computer-aided design/computer-aided manufacturing (CAD/CAM) technologies [4]. Despite their growing popularity, the comparative accuracy of digital versus conventional techniques remains a subject of ongoing investigation, particularly in the context of implant dentistry, where even minor discrepancies can lead to clinical complications such as misfit or peri-implant bone loss [3,5,6]. In a systematic review of 12 studies by Ahmed et al. [3], no significant differences were observed between conventional and digital impressions in terms of accuracy. A study by Atieh et al. [6] reported better accuracy of conventional impressions than optical impressions. In contrast, Mangano et al. [5] reported that digital impressions were more comfortable and acceptable to patients than conventional impressions.

The precision of an impression technique is determined by its ability to accurately replicate the spatial relationship between implants and the surrounding oral structures. Conventional impressions rely on physical materials and laboratory processes scanned using laboratory scanners to create digital models for crown fabrication [2]. By contrast, intraoral scanners directly generate digital datasets, eliminating several intermediate steps and potential sources of error [1]. However, factors such as scanner calibration, soft tissue management, and operator experience can influence the accuracy of digital impressions [3]. Similarly, the marginal fit of crowns, defined as the closeness of the restoration margin to the prepared tooth or implant abutment, plays a pivotal role in ensuring the longevity of the restoration [1]. Poor marginal fit can lead to microleakage, cement dissolution, or secondary caries, compromising both the prosthesis and the supporting implant [7]. A widely recognized methodology for determining the optimal margin gap value has yet to be established. A threshold of less than 120 μm has been documented in a previous study [8], whereas another study has argued that it should be confined to less than 100 μm [9]. Furthermore, it has been postulated that a suitable range for this value is between 20 and 75 μm [7].

Although digital workflows promise enhanced precision through streamlined processes and advanced manufacturing, clinical studies comparing their outcomes with those of conventional methods are essential for validating these claims. Therefore, the need for this study arose from the limited consensus in the literature regarding the superiority of digital versus conventional impression techniques for implant restorations. This study aimed to evaluate and compare the precision of digital and conventional implant impressions and compare the marginal fit of crowns obtained from digital impressions and those obtained from conventional impressions.

## Materials and methods

Study design and setting

This clinical study was designed as a prospective comparative study conducted at the Department of Prosthodontics, Sibar Institute of Dental Sciences, Guntur, India. The duration of the study was from January 2024 to December 2024. Ethical clearance was obtained from the Institutional Ethics Committee (approval number: Pr.188/IEC/SIBAR/2023) before the commencement of the study. This study adhered to the principles outlined in the Declaration of Helsinki (2013 revision). Written informed consent was obtained from all subjects, with detailed information regarding the study objectives, procedures, potential risks, and benefits, to ensure voluntary participation.

Sample size

The sample size was determined using G*Power 3.1.9.2 software (Heinrich Heine University, Düsseldorf, Germany). With an effect size of 0.61 and a power (1-β) of 80%, and 5% level of significance, a sample size of 18 subjects per group was calculated to be sufficient to detect statistically significant differences in the precision of impressions and the marginal fit of crowns between conventional and digital techniques [10].

Eligibility

Inclusion criteria included subjects who had undergone implant placement and were aged >18 years. Subjects with immediately loaded implants, peri-implantitis, or restricted mouth opening were excluded, as these conditions could affect the accuracy of impressions or evaluation of crown fit.

Methodology

Before data collection, the intraoral scanner (3Shape Trios 4, Copenhagen, Denmark) and laboratory scanner (Medit T310 D, Medit Corp., Seoul, South Korea) were calibrated using manufacturer-provided calibration kits to ensure accuracy. Calibration was performed before each scanning session to adjust for potential optical deviations. The stereomicroscope (Magnus MSZ-TR, Magnus Analytics, New Delhi, India) was also calibrated to ensure precise measurements at 40× magnification, with the software (Magvision ×64, Magnus Analytics, New Delhi, India) set to equate 1 pixel to 1 µm for consistency in marginal discrepancy measurements.

Digital impressions were obtained using an intraoral scanner. The scanning process began with the maxillary or mandibular arches, starting with the occlusal surfaces of the molars, followed by the anterior teeth, and extending to the contralateral side of the arch containing the implant abutment. The abutment was scanned thoroughly, followed by the buccal and lingual surfaces of the remaining teeth. This process was repeated three times to get an accurate scan, and the data were saved in Standard Tesselation Language (STL) format. The high-resolution STL files were transferred to the software (Geomagic Control 2014 version 2.0, 3D Systems, Rock Hill, SC, USA) for further analysis.

Conventional impressions were made using a closed-tray implant impression technique with polyvinyl siloxane (PVS) impression material (GC Flexceed, GC Corporation, Tokyo, Japan) with putty and light-body consistency. A perforated dentulous stainless steel stock tray was selected, and the putty material was manipulated and loaded onto the tray. Simultaneously, light-body material was injected around the abutment using a cartridge dispenser (3M ESPE, 3M Company, St. Paul, MN, USA). A single-step impression technique was employed; once set, the impression was removed and inspected for void formation. The abutment was loosened, removed from the implant, and tightened to an implant analog before being reoriented into the impression. The impression was poured with Type IV gypsum (Die Stone, Kalrock, Kalabhai Karson Pvt. Ltd., Mumbai, India) to create a cast, which was checked for voids after the setting. The cast was scanned using a laboratory scanner, and the process was repeated three times. The resulting 3D virtual model was converted to STL format for analysis.

**Figure 1:**
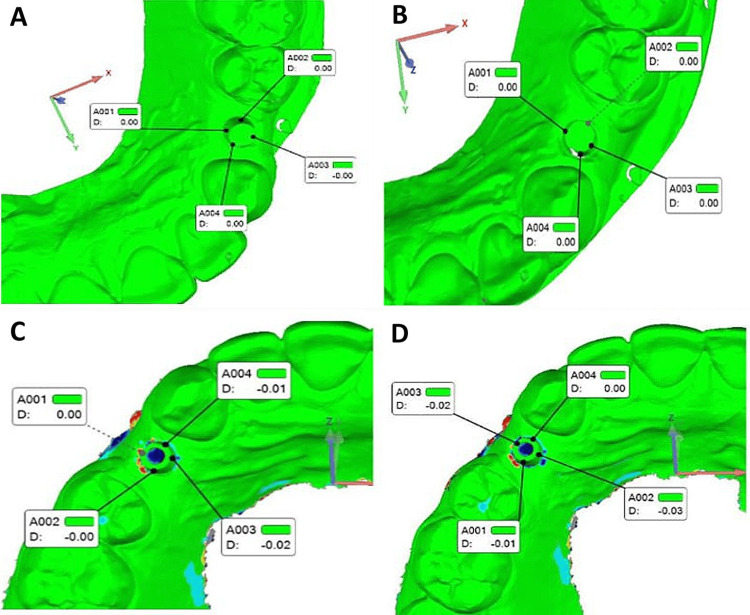
Superimposition analysis of impression scans. (A) Scan 2 superimposed on reference Scan 1 using the laboratory scanner. (B) Scan 3 superimposed on reference Scan 1 using the laboratory scanner. (C) Scan 2 superimposed on reference Scan 1 using the intraoral scanner. (D) Scan 3 superimposed on reference Scan 1 using the intraoral scanner. Color maps showing three-dimensional deviation analysis performed using best-fit alignment in Geomagic Control software. All images are original and correspond to study-specific scan data.

STL datasets from both intraoral and laboratory scanners were imported into the software for analysis. For each scanner, the first STL file served as the reference, whereas the subsequent two files were test files. The scans were superimposed using the best-fit alignment method, and four points on the abutment (buccal, lingual, mesial, and distal) were marked. Deviations at these points were measured to assess precision. After alignment using the best-fit algorithm in Geomagic Control 2014 version 2.0 software, deviations between the reference and test STL files were quantified using the 3D Compare tool to measure differences at the marked points on the abutment, with absolute values averaged to assess precision (Figure 1).

Digital data from both scanners were imported into the Exocad software (Exocad GmbH, Darmstadt, Germany) for the design and fabrication of direct metal laser-sintered crowns. Metal copings were fabricated, followed by ceramic layering. Two cement-retained crowns with occlusal holes were produced for each patient, one from the digital impression and the other from the conventional impression workflow.

The crowns were tested on the implant’s abutment to assess seating and marginal fit. The silicone replication technique was used to evaluate marginal fit. Each crown was filled with light-body PVS material (GC Flexceed) and seated on the abutment. After setting, the crown was removed, leaving a light body on the abutment to represent cement space. The putty material was applied to a light body for stabilization. This process was repeated for both crowns. The replicas were sectioned perpendicularly into two halves in the buccolingual and mesiodistal directions using a BP blade. The margins of each section (mesiobuccal, mesiodistal, distobuccal, and distolingual) were measured under a stereomicroscope at 40× magnification using Magvision ×64 software. The thickness of the light body was recorded in pixels (one pixel = 1 µm).

Statistical analysis

Data were analyzed using SPSS version 20 software (IBM Corp., Armonk, NY, USA). Continuous data from both impression techniques were subjected to the Shapiro-Wilk test and Q-Q plot evaluation to confirm normal distribution, validating the application of parametric tests. Intragroup comparisons across marginal sites were conducted using one-way analysis of variance (ANOVA), whereas intergroup comparisons were performed using the t-test. A p-value of 0.05 was established to determine statistical significance.

## Results

Both groups consisted of nine (50%) males and nine (50%) females, with a mean age of 28.58 ± 4.56 years. Both groups consisted of posterior single-unit implant placement, and there were no drop-outs from the study. Table 1 presents the marginal discrepancy within each group (conventional and digital) at four sites (distobuccal, mesiobuccal, distolingual, and mesiolingual) using a one-way ANOVA. For the conventional group, the mean marginal discrepancies were 109.92 ± 51.26 µm (distobuccal), 103.96 ± 49.99 µm (mesiobuccal), 109.33 ± 54.02 µm (distolingual), and 115.21 ± 51.04 µm (mesiolingual). For the digital group, the corresponding means were 103.65 ± 60.41 µm, 99.55 ± 58.53 µm, 91.96 ± 44.81 µm, and 98.26 ± 53.16 µm, respectively. The results yielded no statistically significant differences in marginal discrepancies across sites within each group (p > 0.05). This suggested that the marginal discrepancies were consistent across the four sites for both conventional and digital methods.

**Table 1:** Intragroup comparison of marginal discrepancy (in µm) within groups using the one-way analysis of variance test. P-values >0.05 denote non-significance. Data are presented as mean and standard deviation (SD).

Site	Conventional (discrepancy in µm)				Digital (discrepancy in µm)			
	Mean ± SD	95% confidence interval for the mean	F-value	P-value	Mean ± SD	95% confidence interval for the mean	F-value	P-value
Distobuccal	109.92 ± 51.26	84.43–135.42	0.18	0.731	103.65 ± 60.41	73.61–133.70	0.14	0.934
Mesiobuccal	103.96 ± 49.99	79.10–128.82			99.55 ± 58.53	70.44–128.66		
Distolingual	109.33 ± 54.02	82.46–136.20			91.96 ± 44.81	69.68–114.25		
Mesiolingual	115.21 ± 51.04	89.83–140.59			98.26 ± 53.16	71.82–124.70		

Table 2 compares the marginal discrepancies between the conventional and digital groups at the same four sites using an independent t-test. A total of 18 (50%) patients were included in each group. For the distobuccal site, the conventional group had a mean discrepancy of 109.92 ± 51.26 µm, while the digital group had a mean discrepancy of 103.65 ± 60.41 µm, with no significant difference (p > 0.05). Similarly, at the mesiobuccal site, means were 103.96 ± 49.99 µm (conventional) and 99.55 ± 58.53 µm (digital), with a p-value of 0.809. For the distolingual site, means were 109.33 ± 54.02 µm (conventional) and 91.96 ± 44.81 µm (digital), with a p-value of 0.301. Lastly, at the mesiolingual site, means were 115.21 ± 51.04 µm (conventional) and 98.26 ± 53.16 µm (digital), with a p-value of 0.336. Across all sites, there were no statistically significant differences in marginal discrepancy between the conventional and digital groups, suggesting comparable performance in terms of marginal fit.

**Table 2:** Intergroup comparison of marginal discrepancy (in µm) between groups using the independent t-test. P-values >0.05 denote non-significance. Data are presented as mean and standard deviation (SD), where N denotes the number of samples in each group.

Site	Groups	N	%	Minimum	Maximum	Range	Mean ± SD	t-value	P-value
Distobuccal	Conventional	18	50%	11.80	214.49	202.69	109.92 ± 51.26	0.34	0.739
	Digital	18	50%	7.70	241.41	233.71	103.65 ± 60.41		
Mesiobuccal	Conventional	18	50%	11.86	205.33	193.47	103.96 ± 49.99	0.24	0.809
	Digital	18	50%	9.54	226.90	217.36	99.55 ± 58.53		
Distolingual	Conventional	18	50%	15.31	221.86	206.55	109.33 ± 54.02	1.05	0.301
	Digital	18	50%	12.61	158.45	145.84	91.96 ± 44.81		
Mesiolingual	Conventional	18	50%	18.55	193.40	174.85	115.21 ± 51.04	0.98	0.336
	Digital	18	50%	10.98	181.28	170.30	98.26 ± 53.16		

Table 3 shows the precision between the conventional and digital groups using an independent t-test. The conventional group had a mean precision of 0.00 ± 0.00 µm, indicating no variability, whereas the digital group had a mean precision of 1.81 ± 17.91 µm, indicating greater variability. No statistically significant difference in precision was observed between the two groups. This suggested that both methods achieved similar levels of precision, despite the digital group showing more variability in its measurements.

**Table 3:** Intergroup comparison of precision (in µm) between groups using the independent t-test. P-values >0.05 denote non-significance. Precision was assessed as the mean absolute deviation (in µm) between repeated scans using best-fit alignment in Geomagic Control software. Data are presented as mean and standard deviation (SD), where N denotes the number of samples in each group.

Precision	N	Mean	SD	t-value	P-value
Conventional	18	0.00	0.00	0.426	0.672
Digital	18	1.81	17.91		

## Discussion

This study evaluated the precision of implant impressions and the marginal fit of crowns fabricated using conventional and digital impression techniques. The study found no statistically significant differences between the two methods in terms of marginal discrepancy. The results of this study indicated no significant differences in marginal discrepancies across the four measured sites within both the conventional and digital groups. The values were within the clinically acceptable threshold of 120 µm, which is considered adequate to prevent microleakage and peri-implant complications [8]. Consistency across sites suggested that both techniques produced a uniform marginal fit, which is critical for long-term implant success.

Intergroup comparisons revealed no significant differences between the conventional and digital groups, with mean discrepancies slightly lower in the digital group at most sites. This aligns with the findings of Ahlholm et al. [11], who conducted a systematic review and reported comparable marginal accuracies between intraoral scanners and conventional impressions of single-unit restorations. Similarly, Chochlidakis et al. [12] found no significant differences in marginal fit for single-implant crowns, with mean discrepancies ranging from 80 to 120 µm for both methods, which is consistent with the results of the present study.

However, other studies have reported contrasting results. For instance, Mangano et al. [5] observed that digital impressions using intraoral scanners, such as 3Shape Trios, provided superior marginal fit compared to conventional methods in multi-unit restorations, attributing this to reduced material distortion. The lack of significant differences in the present study may be due to its focus on single-unit restorations, which are less susceptible to cumulative errors than multi-unit cases. Additionally, the use of high-resolution scanners (3Shape Trios 4) and precise measurement techniques (stereomicroscopy at 40× magnification) likely minimized discrepancies, contributing to the equivalence between methods.

The slightly higher standard deviations in the digital group suggested greater variability, possibly due to factors such as operator technique, patient movement, or environmental conditions such as saliva interference. Ender and Mehl [13] noted that intraoral scanning accuracy can be affected by operator experience and scanning conditions, which may explain the observed variability. The lower variability in the conventional group could be attributed to the standardized closed-tray technique and the use of Type IV gypsum, which provides stable and reproducible casts.

Our results showed no significant difference in precision between the conventional (0.00 ± 0.00 µm) and digital (1.81 ± 17.91 µm) groups. The zero variability in the conventional group indicated highly reproducible impressions, likely due to the controlled laboratory scanning of gypsum casts using the Medit T310 D scanner. The digital group’s higher standard deviation suggests variability in intraoral scanning, potentially due to challenges such as soft tissue interference or scan alignment errors in the Geomagic Control 2014 software.

These findings are supported by Ender and Mehl [13], who reported that modern intraoral scanners achieve precision comparable to that of laboratory scanners for single-unit restorations with deviations typically below 10 µm. Similarly, Imburgia et al. [14] found that intraoral scanners, such as the 3Shape Trios, offer precision within 5-10 µm for single implants, aligning with the digital group mean of 1.81 µm. However, contrasting studies, such as that by Seelbach et al. [15], undertook an in vivo investigation to assess the viability and accuracy of the digital scanning methodologies. Ten complete arch intraoral scans were performed using the iTero CAD/CAM system along with ten conventional impregnum impressions, all sourced from a single patient. The results revealed that iTero scans from the patient demonstrated the lowest level of precision.

The equivalence in this study might be attributed to the use of a single-unit restoration model, which reduces the complexity of the scan stitching and alignment errors inherent in multi-unit cases. The calibration of both scanners before each session and the use of a best-fit alignment algorithm in Geomagic Control 2014 likely minimized systematic errors, contributing to comparable precision. However, the variability of the digital group underscores the importance of standardized scanning protocols and operator training, as highlighted by Richert et al. [16], who emphasized the learning curve associated with intraoral scanners.

Several factors likely contributed to the lack of significant differences between the two techniques. First, the use of advanced technology, such as the 3Shape Trios 4 intraoral scanner and Medit T310 D laboratory scanner, ensured high accuracy in both workflows [17]. In contrast, Natsubori et al. [18] reported greater accuracy with laboratory scanners than intraoral scanners. Second, the study’s focus on single-unit restorations minimized errors associated with scan stitching or impression material distortion, which are more pronounced in multi-unit or full-arch cases [5].

The silicone replication technique and stereomicroscopy provided precise measurements and reduced errors in the marginal discrepancy assessment. The calibration of the Magnus MSZ-TR stereomicroscope to equate 1 pixel to 1 µm ensured high resolution, enhancing the reliability of the results. Additionally, the standardized protocols for both impression techniques, including the use of PVS for conventional impressions and Exocad for crown design, likely contributed to the consistency observed across the groups.

The lack of significant differences might also reflect the clinical acceptability of these two methods. Marginal discrepancies below 120 µm and precision deviations below 10 µm are considered adequate for implant restorations [8,13]. The slightly higher variability in the digital group could be due to operator-dependent factors, such as scanning technique, or patient-related factors, such as saliva or soft tissue movement, which are less prevalent in the controlled laboratory scanning of conventional casts.

Although the study demonstrated clinically acceptable results with no significant differences between groups, several methodological limitations should be noted. The sources of error within each workflow, such as those arising from impression materials, stone pouring, or scanner variability, were not independently assessed. This lack of disaggregated analysis may overstate the reproducibility of the digital workflow. Additionally, standard implant transfer protocols using multiple impression or scan bodies were not employed, as the study aimed to reflect typical clinical procedures rather than idealized or experimental conditions. These factors should be considered when interpreting the results, and future studies are encouraged to isolate individual sources of error to more precisely characterize workflow accuracy.

Clinical implications

The comparable performance of conventional and digital impression techniques suggests that clinicians can choose one method based on practical needs, costs, and patient preferences. Digital impressions offer advantages such as reduced patient discomfort, faster workflows, and integration with CAD/CAM systems, which enhance efficiency. However, conventional impressions remain a reliable and cost-effective option, particularly in settings with limited access to digital technology. Equivalence in marginal fit and precision supports the adoption of digital workflows for single-unit implant restorations, provided that clinicians are trained in proper scanning techniques.

Limitations and future directions

The limitations of this study included its small sample size (18 subjects per group), which, although statistically sufficient, might not capture the full range of clinical variability. Larger studies are required to confirm these subtle differences. The focus on single-unit restorations limited their generalizability to multi-unit or full-arch cases, where digital impressions might face challenges. Operator experience was not controlled, potentially influencing digital impression variability. Future studies should standardize the operator training and explore complex restorations. The silicone replication technique, while precise, involved manual processes that could introduce errors, and automated 3D measurement methods could improve accuracy. Long-term clinical outcomes, such as peri-implant health, were not assessed and should be investigated in future longitudinal studies.

## Conclusions

The findings of the study revealed no statistically significant differences in marginal discrepancy or precision between the two methods, indicating comparable performance. Both techniques produced results within clinically acceptable limits, supporting the reliability of conventional impressions and clinical viability of digital workflows. Digital impressions offer advantages, such as improved patient comfort and integration with CAD/CAM systems, whereas conventional methods remain dependable, particularly in resource-limited settings. Clinicians can choose either approach based on their practical needs or expertise. However, the study’s focus on single-unit restorations and limited sample size suggests the need for further research into multi-unit restorations and long-term outcomes to confirm the broader applicability of these findings.
